# Calcitonin Gene-Related Peptide Regulates Type IV Hypersensitivity through Dendritic Cell Functions

**DOI:** 10.1371/journal.pone.0086367

**Published:** 2014-01-21

**Authors:** Norihisa Mikami, Kaori Sueda, Yusuke Ogitani, Ippei Otani, Miku Takatsuji, Yasuko Wada, Keiko Watanabe, Rintaro Yoshikawa, Satoshi Nishioka, Nagisa Hashimoto, Yayoi Miyagi, So-ichiro Fukada, Hiroshi Yamamoto, Kazutake Tsujikawa

**Affiliations:** Laboratory of Molecular and Cellular Physiology, Graduate School of Pharmaceutical Sciences, Osaka University, Osaka, Japan; Istituto Superiore di Sanità, Italy

## Abstract

Dendritic cells (DCs) play essential roles in both innate and adaptive immune responses. In addition, mutual regulation of the nervous system and immune system is well studied. One of neuropeptides, calcitonin gene-related peptide (CGRP), is a potent regulator in immune responses; in particular, it has anti-inflammatory effects in innate immunity. For instance, a deficiency of the CGRP receptor component RAMP 1 (receptor activity-modifying protein 1) results in higher cytokine production in response to LPS (lipopolysaccharide). On the other hand, how CGRP affects DCs in adaptive immunity is largely unknown. In this study, we show that CGRP suppressed Th1 cell differentiation via inhibition of IL-12 production in DCs using an *in vitro* co-culture system and an *in vivo* ovalbumin-induced delayed-type hypersensitivity (DTH) model. CGRP also down-regulated the expressions of chemokine receptor CCR2 and its ligands CCL2 and CCL12 in DCs. Intriguingly, the frequency of migrating CCR2^+^ DCs in draining lymph nodes of RAMP1-deficient mice was higher after DTH immunization. Moreover, these CCR2^+^ DCs highly expressed IL-12 and CD80, resulting in more effective induction of Th1 differentiation compared with CCR2^−^ DCs. These results indicate that CGRP regulates Th1 type reactions by regulating expression of cytokines, chemokines, and chemokine receptors in DCs.

## Introduction

Accumulating evidence has demonstrated that the immune system is tightly regulated by nervous system-derived mediators such as hormones, cytokines, and neurotransmitters [1], [2]. Calcitonin gene-related peptide (CGRP), a 37-amino acid neuropeptide, is one of the potent modulators connecting the nervous and immune systems. CGRP is produced in the neural body of dorsal root ganglion (DRG) cells, is released from the sensory nerve endings, and mainly acts as a pain transmitter and vasodilator *in vivo*. There are two isoforms of CGRP: α and β in rats and mice and I and II in humans. Both CGRP isoforms bind to a specific receptor composed of receptor activity-modifying protein 1 (RAMP1) and calcitonin receptor-like receptor (CLR) [3]. Because CLR couples Gαs protein, the binding of CGRP to its receptor increases the levels of intracellular cAMP [4], [5]. CGRP receptors are also expressed on immune cells, and CGRP modulates immune responses through its receptor binding. Our previous studies using RAMP1-deficient mice showed that CGRP physiologically up-regulates IL-4, IL-9, and IL-17 productions by CD4^+^ Th cells [6]–[9]. On the other hand, CGRP inhibits the production of proinflammatory cytokines after lipopolysaccharide (LPS) administration to mice [10]. These data strongly suggest that CGRP is a potent regulator of both innate and adaptive immune reactions.

Dendritic cells (DCs) regulate and link both innate and adaptive immune responses. In this process, costimulatory molecules and antigen presentation are essential for T cell activation [11], [12]. The differentiation of naïve Th cells is also partially regulated by DCs. In particular, Th1 cell differentiation is mainly driven by DC-derived IL-12 [13], and the interaction between Th cells and DCs via CD40/CD40L is a key process for the production of IL-12 at the stage of antigen presentation [14]. However, the effect of CGRP on the function of DCs during Th cell differentiation is largely unknown, although the direct effects of CGRP on Th cells are well studied.

While DCs are involved in the defense against infections, some allergic reactions are caused by accelerated immune responses that arise from DCs. Delayed type hypersensitivity (DTH) is one of the type IV hypersensitivity, like the tuberculin reaction, and it is mediated by Th1 cells or CD8^+^ T cells. Generally, adaptive immunity to the specific antigen is critical in this response; therefore, the antigen-presenting ability and/or cytokine production of DCs affects the progression of DTH. CGRP inhibits Th1 cell differentiation via a direct effect on Th cells, but its physiological roles in DCs are still poorly understood.

In this study, we demonstrate that CGRP inhibits not only the production of inflammatory cytokines but also the expression of chemokine receptors through activation of the cAMP/PKA pathway in DCs. We also show that CGRP physiologically regulates IL-12 production in DCs after ovalbumin (OVA) immunization and inhibits Th1 cell differentiation. Moreover, CGRP suppresses OVA-induced DTH responses through these anti-inflammatory effects. These results indicate that CGRP inhibits Th1-mediated immune response through the cytokine production and function of both DCs and Th cells, and these findings will lead to a deeper understanding of DC functions and immune regulation by CGRP.

## Methods

### Mice

BALB/c mice (6–10 weeks old) were purchased from Charles River Japan (Kanagawa, Japan). RAMP1-deficient mice were generated and heterozygous knockout mice were backcrossed to BALB/c mice for 12 generations [10]. OVA-specific TCRαβ transgenic mice (DO11.10 Tg) were kindly contributed by Hiromichi Ishikawa (Keio University, Tokyo, Japan). All procedures involving animals were approved by the Experimental Animal Care and Use Committee of the Graduate School of Pharmaceutical Sciences, Osaka University.

### Antibodies and Reagents

FITC-conjugated and biotin-conjugated anti-mouse IFN-γ monoclonal antibody (mAb) (XMG1.2), purified anti-mouse IFN-γ mAb (R4-6A2), FITC-conjugated anti-mouse CD3 mAb (2C11), FITC-conjugated or biotin-conjugated anti-mouse CD11c mAb (HL3), FITC-conjugated anti-mouse CD19 mAb (1D3), PE-conjugated anti-mouse CD25 mAb (PC61) and allophycocyanin-conjugated streptavidin were purchased from BD Pharmingen (San Diego, CA). APC-Cy7-conjugated anti-mouse CD4 mAb (GK1.5) and FITC-conjugated anti-mouse CD62L mAb (MEL-14) were purchased from BioLegend (San Diego, CA). Biotin-conjugated anti-MHC class II (I-A/I-E) (M5/114.15.2), PE-conjugated anti-mouse CD45RB mAb (C363.16A), PE-conjugated anti-mouse CD80 mAb (16-10A1), PE-conjugated anti-mouse CD86 mAb (GL1), and PE-Cy5-conjugated anti-mouse CCR7 (4B12) were purchased from eBioscience (San Diego, CA). Allophycocyanin-conjugated anti-mouse CCR2 mAb (475301) was purchased from R&D Systems (Minneapolis, MN), and PE-conjugated streptavidin was purchased from Dako (Carpinteria, CA). Anti-mouse IL-12 mAb (C15.6) was prepared from the culture supernatant of hybridoma cells. Adjuvant incomplete Freund (IFA), adjuvant complete Freund (CFA), and dibutyryl cAMP (db-cAMP) were purchased from Wako Pure Chemical (Osaka, Japan). N^6^-benzoyl-cAMP (6-bnz-cAMP) and 8-(4-chlorophenylthio)-2′-O-methyl-cAMP (8-CPT-cAMP) were purchased from Calbiochem (San Diego, CA). LPS, PKA inhibitor H89, and phosphodiesterase inhibitor IBMX (3-isobtyl-1-methylxanthine) were purchased from Sigma Aldrich (St. Louis, MO). αCGRP was purchased from Peptide Institute (Osaka, Japan), and βCGRP was purchased from Phoenix Pharmaceuticals, Inc. (Burlingame, CA). LPS-free ovalbumin (OVA) was purchased from Seikagaku Corp. (Tokyo, Japan). Anti-mouse IL-12 mAbs (C17.8.20 and C15.6.7) were obtained from the supernatant of hybridomas, and C15.6.7 was used after biotinylatination as the second antibody in ELISA.

### DC Isolation and Stimulation

**Figure 1:**
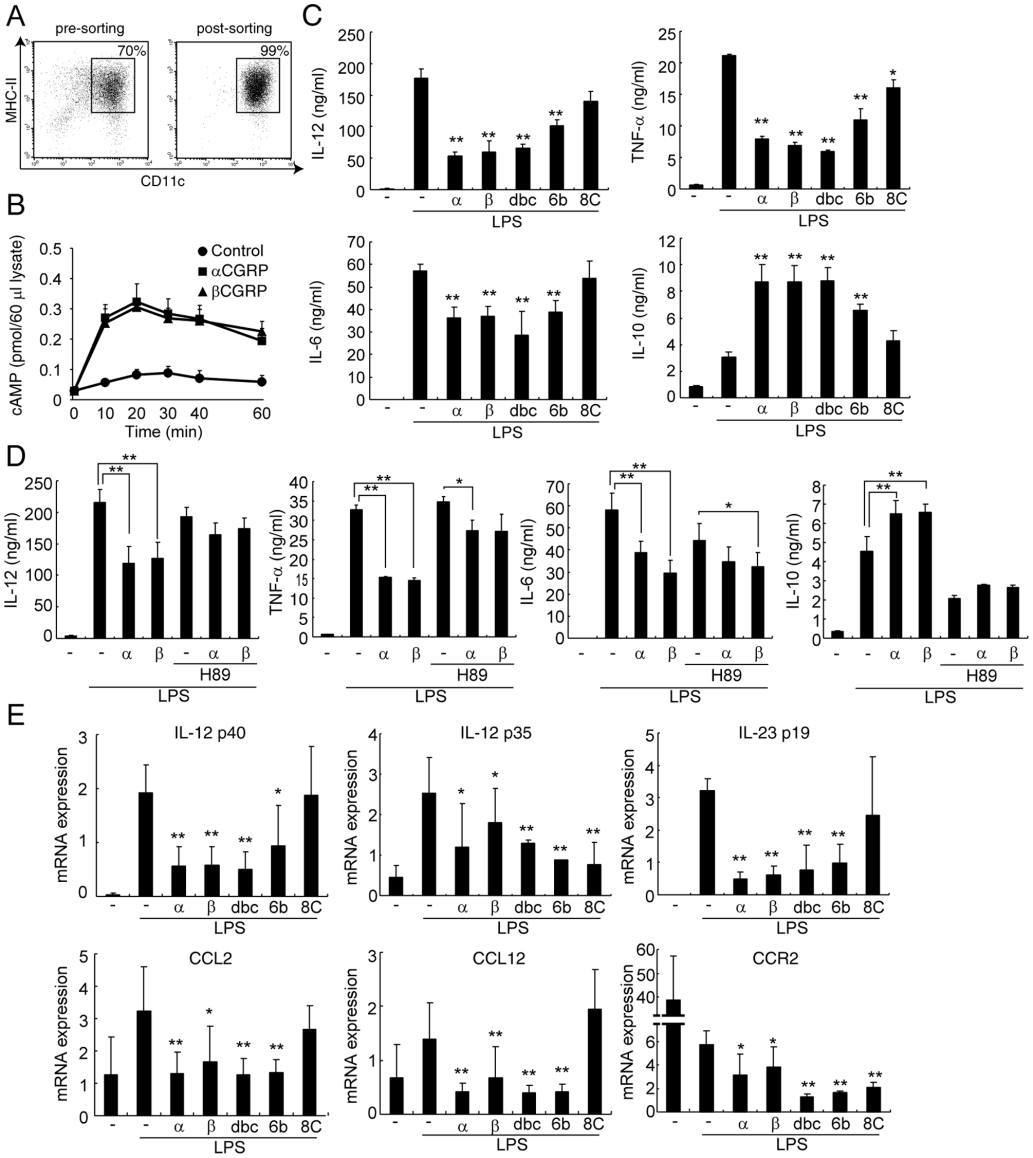
CGRP affects cytokine productions in DCs via cAMP/PKA pathway. (A) The percentage of CD11c^+^ BMDCs was analyzed by FACS. (B) BMDCs were incubated with or without CGRP in the presence of LPS stimulation. The cellular cAMP levels were determined after the indicated time periods. Values are presented as mean ± SD (n = 3). (C) The concentrations of each cytokine were determined by ELISA after 24 h stimulation with LPS. Values are presented as mean ± SD (n = 3). *P<0.05, **P<0.01 VS. LPS only. (D) The concentrations of each cytokine were determined by ELISA after 24 h stimulation with LPS. Values are presented as mean ± SD (n = 3). *P<0.05, **P<0.01. (E) The mRNA expression was determined by real-time PCR after 6 h stimulation with LPS. Values are presented as mean ± SD (n = 3). *P<0.05, **P<0.01 vs. LPS only. α: αCGRP (1 nM), β: βCGRP (1 nM), dbc: db-cAMP (100 µM), 6b: 6-bnz-cAMP (100 µM), and 8C: 8-CPT-cAMP (100 µM) were added at the start of stimulation.

Bone marrow-derived dendritic cells (BMDCs) were generated *in vitro* as described [10]. Briefly, the bone marrow cells were cultured in complete medium containing mouse granulocyte-macrophage colony-stimulating factor (20 ng/ml) for 10 days. CD11c^+^ cells were sorted by a FACSAria II cell sorter as described in Fig. 1A. The BMDCs were stimulated with LPS (0.1 µg/ml) or OVA (200 µg/ml)+anti-CD40 mAb (5 µg/ml) in the presence or absence of CGRP (1 nM). The intracellular cAMP concentration was measured using a cAMP Screen Assay kit (Applied Biosystems, Carlsbad, CA).

Spleen-derived DCs were sorted as CD11c^high^ cells using a FACSAria II cell sorter as previously described [7].

### Isolation of Naïve Th Cells and Co-culture System

CD4^+^CD25^−^CD44^low^CD62L^+^ naïve T cells were purified from splenocytes using an FACSAria II as previously described [8]. Purified naive Th cells were cocultured with BMDCs or spleen DCs (10∶1 ratio) in the presence of 200 µg/ml OVA for 72 h. After stimulation, the cells and supernatants were used in the following experiments.

### Detection of Cytokines by Intracellular Staining and ELISA

For cytokine staining, cocultured Th cells were restimulated by 15 ng/ml phorbol 12-myristate 13-acetate and 750 ng/ml ionomycin. After 6 h restimulation, those cells were fixed and stained using a BD Cytofix/Cytoperm kit according to the manufacturer's instructions.

IL-12 and IFN-γ concentrations in cell supernatants were measured as previously described [7], [10]. IL-6, IL-10, and TNF-α concentrations in cell supernatants were measured using a Mouse ELISA MAX SET Standard (BioLegend).

### RNA Extraction and Real-time RT-PCR

Total RNA was extracted using TRIzol reagent (Invitrogen, Carlsbad, CA). Total RNA was reverse transcribed into cDNA with a PrimeScript RT reagent kit (TaKaRa, Kyoto, Japan) according to the manufacturer's instructions. The PCR primers used were as follows: IL-10 forward, 5′-CTGAGGCGCTGTCATCGATT-3′; IL-10 reverse, 5′-AGGTCCTGGAGTCCAGCAGA-3′; IL-12 p40 forward, 5′-ACATCAAGAGCAGTAGCAGTTCC-3′; IL-12 p40 reverse, 5′-CAGTTGGGCAGGTGACATCC-3′; IL-12 p35 forward, 5′-CTGGAACTACACAAGAACGAGAG-3′; IL-12 p35 reverse, 5′-CTTCAAGTCCTCATAGATGCTACC-3′; IL-23 p19 forward, 5′-CAGCAGCTCTCTCGGAAT-3′; IL-23 p19 reverse, 5′-ACAACCATCTTCACACTGGATTACG-3′; CCL2 forward, 5′-CCTGGATCGGAACCAAATGA-3′; CCL2 reverse, 5′-CGGGTCAACTTCACATTCAAAG-3′; CCL12 forward, 5′-GGGAAGCTGTGATCTTCAGG-3′; CCL12 reverse, 5′-GGGAACTTCAGGGGGAAATA-3′; CCR2 forward, 5′-AGGCCATGCAGGTGACAG-3′; CCR2 reverse, 5′-AGGCAAACTGTCACTTACT-3′; glyceraldehyde-3-phosphate dehydrogenase (GAPDH) forward, 5′-CATGCCATCACTGCCACCC-3′; and GAPDH reverse, 5′-GGTAGGAACACGGAAGGCC-3′. Quantitative RT-PCR was performed using a Lightcycler system (Roche, Basel, Switzerland).

### OVA-induced DΤH Model

Mice were sensitized by subcutaneously injecting 100 µg OVA in 100 µl complete Freund's adjuvant (CFA) at the base of the tail. Seven days after sensitization, mice were challenged with injection of 200 µg heat-aggregated OVA/50 µl CFA into the right ear and 50 µl PBS into the left ear. For BMDCs-induced DTH responses, WT or RAMP1-deficient BMDCs were pulsed with 200 µg/ml OVA for 6 hr. One million pulsed DCs were injected subcutaneously at the base of the tail of BALB/c mice. Seven days after sensitization, 200 µg OVA in 50 µl IFA was injected into the right footpad and IFA in 50 µl PBS was injected into the left footpad. Ear and footpad swellings were measured using a dial thickness gauge (Peacock, Osaka, Japan).

For detection of serum OVA-specific IgG, diluted serum were incubated on the plates coated with 1 µg/ml OVA. Horseradish peroxidase-labeled goat anti-mouse total IgG, IgG1, IgG2a, IgG2b or IgG3 (Zymed laboratories, San Francisco, CA) were used as secondary antibodies.

### Statistical Analysis

Statistical significance between two groups was assessed using unpaired Student's *t* test. To compare more than two groups, non-repeated measures analysis of variance (ANOVA) followed by Student–Newman–Keuels test were used. A probability of less than 5% (*p*<0.05) or 1% (*p*<0.01) was considered statistically significant.

## Results

### CGRP Regulates Cytokine Production From DCs Through cAMP/PKA Pathway

To investigate the effects of CGRP on DCs, purified bone marrow-derived dendritic cells (BMDCs) were prepared (Fig. 1A) and stimulated by several TLR ligands. Consistent with our previous report [10], CGRP down-regulated IL-12 and TNF-α productions from TLR-stimulated BMDCs (Fig. S1). CGRP increases the intracellular level of cAMP in BMDCs (Fig. 1B). Therefore, the roles of the cAMP/PKA pathway were examined to clarify the CGRP-mediated regulation of these cytokine productions. In addition, several studies have revealed that activation of the cAMP/PKA pathway inhibits IL-12 and TNF-α productions in APCs [15]–[17]. Therefore, we first examined the effects of cAMP analogs on the production of IL-12, TNF-α, IL-6, and IL-10 in BMDCs stimulated by LPS. As shown in Fig. 1C, a cAMP analog (db-cAMP) inhibited the production of the pro-inflammatory cytokines IL-12, TNF-α and IL-6 as well as CGRP did. Moreover, similar effects were also observed when a PKA-specific cAMP analog (6-bnz-cAMP) was used in BMDCs stimulated by LPS, although exchange protein directly activated by cAMP (Epac)-specific cAMP analog (8-CPT-cAMP) did not. On the other hand, IL-10 production was up-regulated by CGRP or cAMP analogs (Fig. 1C). Because IL-10 is one of the potent immunosuppressive cytokines, these results suggest that CGRP signaling works as an anti-inflammatory molecule in DCs.

To further clarify the roles of PKA in CGRP-mediated cytokine regulation in BMDCs, a PKA inhibitor was added to BMDCs cultured with both LPS and CGRP. As shown in Fig. 1D, in the presence of PKA inhibitor H89, CGRP has little or no effect on IL-12, TNF-α, IL-6, and IL-10 production by BMDCs (Fig. 1D). Therefore, these results suggest that cAMP/PKA contributes to the cytokine regulation of CGRP in DCs.

The transcriptional regulation by CGRP was also examined. In an ELISA experiment, the amounts of IL-12 p70 heterodimer were elucidated. IL-12 p70 is composed of p35 and p40 subunits, and the p40 subunit can also combine with the p19 subunit to form IL-23, which expands the number of Th17 cells. Therefore, mRNA expressions of p40, p35, and p19 were quantified by real-time PCR. As shown in Fig. 1E, CGRP and PKA activator inhibited transcription of these IL-12 subunits. These results imply that CGRP suppressed not only Th1 but also Th17 differentiation because IL-6 and IL-23 are critical factors in the differentiation and proliferation of Th17 cells. However, we previously demonstrated that CGRP physiologically promotes Th17 responses [8]. In order to explicate this discrepancy, we further analyzed the effects of CGRP on the co-culture assay of Th0 and BMDCs in the presence of TGF-β. In the presence of TGF-β, we detected IL-17 production in the co-culture supernatant and also observed that CGRP increased the production of IL-17 from Th17 cells (Fig. S2). This result suggests that the direct promoting effect of CGRP on Th17 cells, rather than through DCs, is more important for enhanced Th17 response by CGRP although CGRP inhibits IL-6 production from DCs. In addition, the inhibitory effect of CGRP on IL-12 production may contribute to the increased development of Th17 cells because IL-12 strongly suppresses its differentiation. Therefore, we concluded that CGRP does not have an inhibitory effect on DCs in Th17 cell differentiation.

In addition, the mRNA expressions of TNF-α and IL-10 were also regulated by CGRP (Fig. S3). Interestingly, the expression of IL-10 was activated by CGRP even without LPS stimulation (Fig. S3). Furthermore, Epac-specific activator also down-regulated the IL-12 p35 subunit (Fig. 1E). These data show that CGRP and the cAMP/PKA pathway regulate cytokine production on the transcriptional level, although we cannot exclude the possibility that other mechanisms, including Epac pathway, regulate CGRP-mediated cytokine production.

To further elucidate the regulatory mechanisms of CGRP on DC function, we performed real-time PCR array analyses using the mRNA of BMDCs treated with LPS, LPS+αCGRP, or LPS+βCGRP (Fig. S4). In these experiments, we found that CGRP regulates the chemokine receptor CCR2 and CCL12, which is one of its ligands. Recently, using CCR2-deficient mice, it was shown that CCR2 enhances DC maturation, IL-12 production, and Th1 response [18]–[20]. Therefore, the effect on CCR2 signaling might be one of the mechanisms in the regulation of CGRP in DC functions. In order to investigate the roles of CGRP in CCR2 expression, we first examined the expression of CCR2 and CCL12 by real-time PCR. As shown in Fig. 1E, CGRP down-regulated the mRNA expressions of CCR2 and CCL12 in BMDCs stimulated by LPS, which is consistent with the results of real-time PCR array analyses (Fig. 1E). In addition, db-cAMP and 6-bnz-cAMP showed similar effects (Fig. 1E). Moreover, CGRP, db-cAMP, and 6-bnz-cAMP inhibited the expression of CCL2, which is another CCR2 ligand (Fig. 1E). CCR2-mediated signaling is essential for the maturation of DCs; therefore, these results suggest that CGRP and activation of the cAMP/PKA pathway inhibit DC maturation, which is regulated by CCL2/CCL12 and CCR2 pathways.

The results of gene expression analyses indicated that CGRP inhibits both CCR2 and its ligands in DCs. However, it is still unknown whether DC-derived CCL2 and CCL12 have physiological functions, including autocrine effects. In general, monocytes/macrophages are the major source of CCL2 [21]. Therefore, CCL production by both BMDCs and bone marrow-derived macrophages (BMMs) was examined. We found that BMMs produced larger amounts of CCL2 and CCL12 than BMDC, and CGRP inhibited CCL2 and CCL12 production in BMMs as well (Fig. S5). These results suggest that CGRP may have some suppressive functions in the CCL2/CCL12 and CCR2 signaling pathways in DCs by affecting both autocrine and paracrine pathways although the physiological importance of CCL2 and CCL12 derived from DCs remains unknown.

### CGRP suppresses Th1 differentiation through a direct effect on both DCs and Th cells

**Figure 2:**
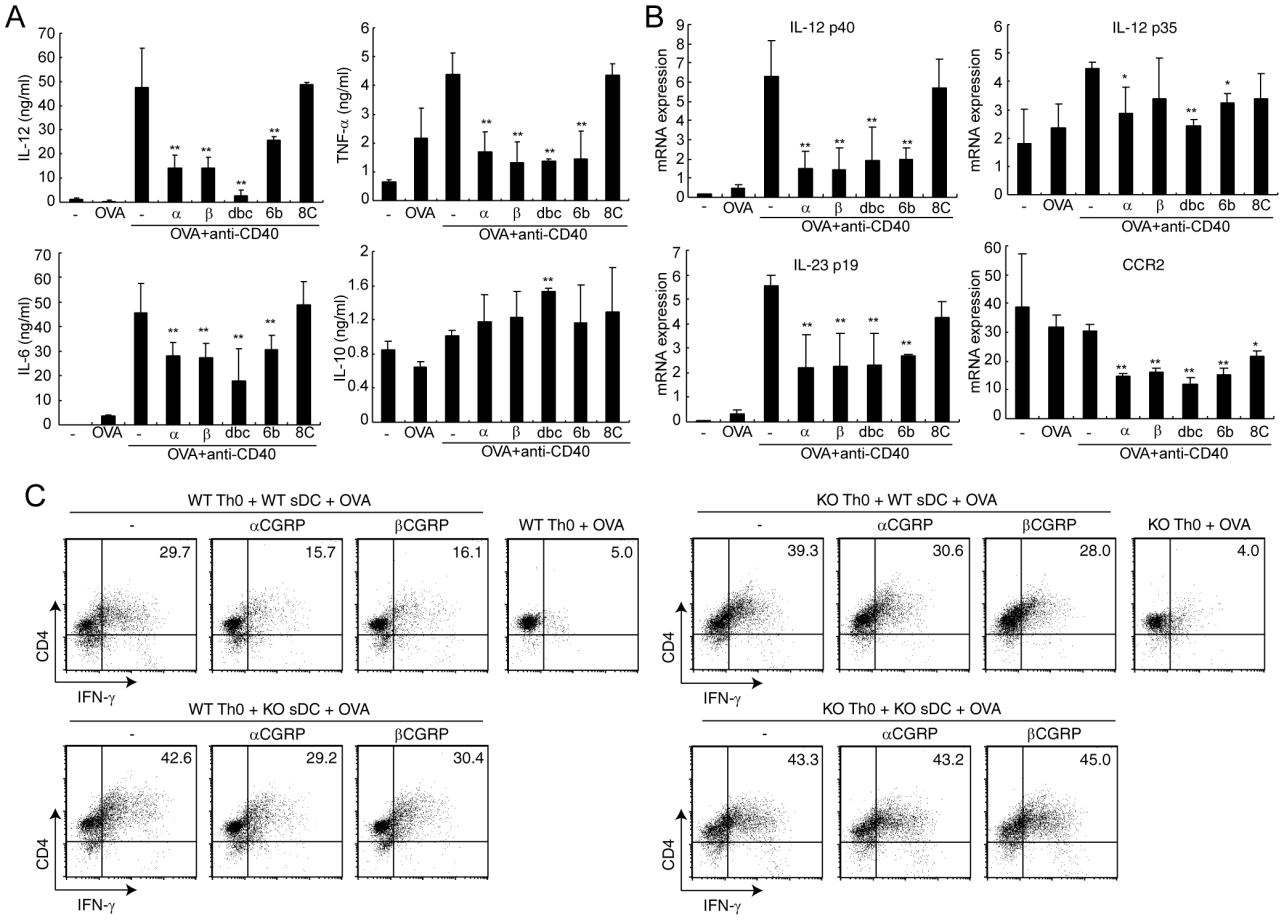
CGRP affects cytokine production in DC-Th cell interaction. (A) The concentrations of each cytokine were determined by ELISA after 24 h stimulation with OVA and anti-CD40 mAb. Values are presented as means ± SD (n = 3). **P<0.01 VS. OVA+anti-CD40 mAb. (B) The mRNA expression was determined by real-time PCR after 6 h stimulation with OVA and anti-CD40 mAb. Values are presented as mean ± SD (n = 3). *P<0.05, **P<0.01 vs. OVA+anti-CD40 mAb. (C) BMDCs and DO 11.10 Th0 cells were co-cultured in the presence of 200 µg/ml OVA for 72 h. IFN-γ production was detected by FACS after intracellular staining. Data are represented of three independent experiments. α: αCGRP (1 nM), β: βCGRP (1 nM), dbc: db-cAMP (100 µM), 6b: 6-bnz-cAMP (100 µM), and 8C: 8-CPT-cAMP (100 µM) were added from start point of stimulation.

Next we investigated whether CGRP affects DCs in the immunological synapse. It is known that CD40-CD40L interaction plays an essential role in DC activation and IL-12 production in this immunological contact between DCs and T cells [13]. Therefore, BMDCs were stimulated with anti-CD40 mAbs and OVA in order to imitate the interaction of DCs and DO11.10 T cells. Like LPS-stimulated BMDCs, CGRP and the cAMP/PKA pathway significantly inhibited IL-12, TNF-α, and IL-6 production in BMDCs stimulated with anti-CD40 mAbs (Fig. 2A). Furthermore, mRNA expression of IL-12, IL-23, and CCR2 were also down-regulated by CGRP (Fig. 2B). However, IL-10 production was not induced by this stimulatory condition and no effect of CGRP was observed (Fig. 2A).

Because it was expected that CGRP inhibits Th1 differentiation by regulating IL-12 production, we performed co-culture experiments using spleen DCs and naïve DO11.10 Th cells. When DCs were prepared from WT mice, CGRP inhibited Th1 differentiation from Th0 cells in both WT- and RAMP1-deficient Th0 cells (Fig. 2C). CGRP did not suppress Th1 differentiation when both types of cells were derived from RAMP1-deficient mice, but Th1 differentiation was also inhibited by CGRP in a co-culture of WT-Th0 cells and RAMP1-deficient DCs (Fig. 2C). Therefore, these results indicated that CGRP has the ability to suppress Th1 differentiation through a direct effect on both DCs and Th cells in receptor-dependent manner.

### CGRP inhibits DTH and Th1 Response In vivo

**Figure 3:**
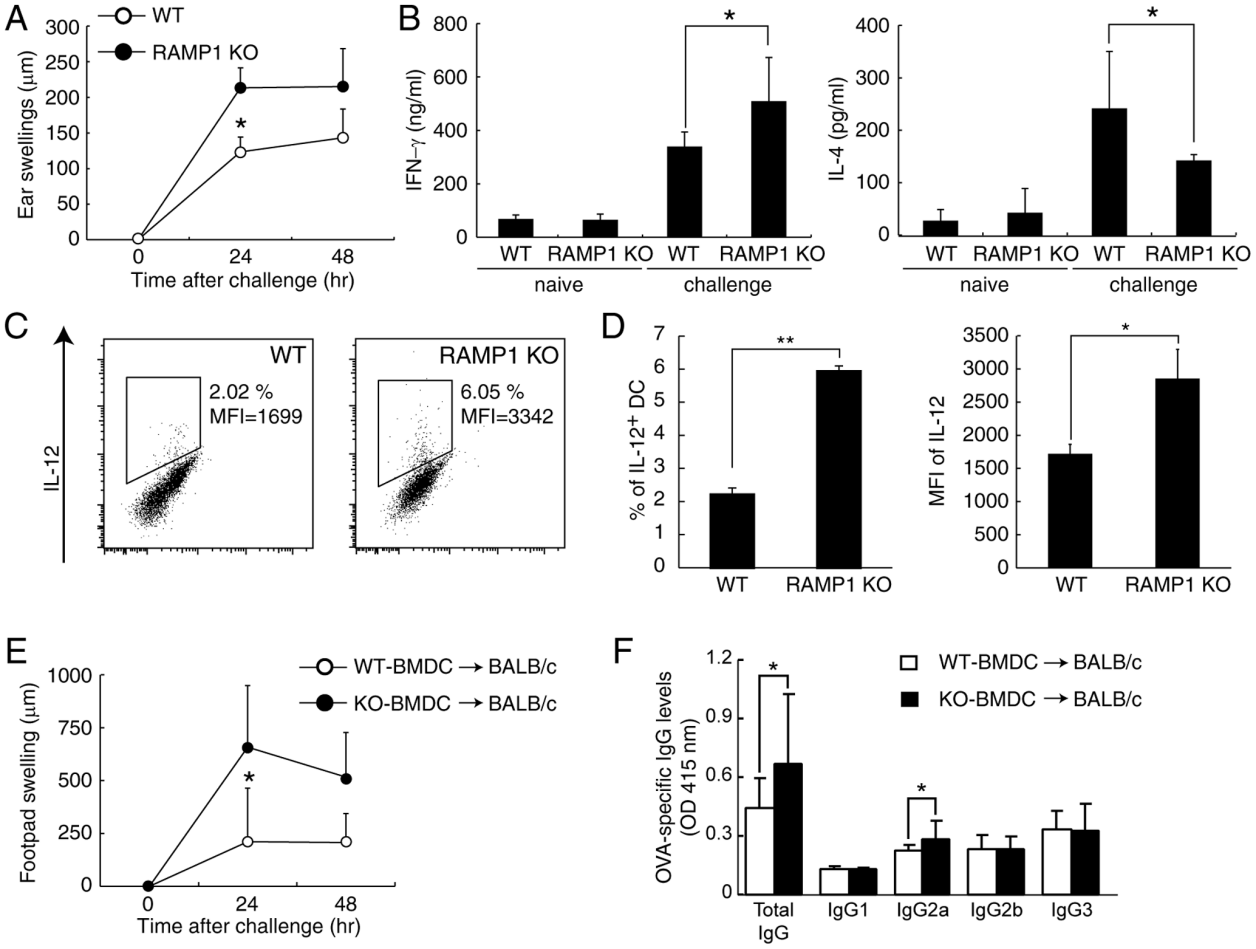
CGRP physiologically inhibits DTH response. (A) WT and RAMP1-deficient mice were sensitized with OVA, and ear swelling was measured after challenge. Values are represented as mean ± SD (n = 9). (B) Draining LNs were collected 24 h after challenge, and total lymphocytes were stimulated with anti-CD3 and anti-CD28 mAbs for 48 h. IL-4 and IFN-γ concentrations in the supernatants were measured by ELISA. Values are presented as means ± SD (n = 6). *P<0.05. (C) CD11c^+^ DCs were purified from dLNs 96 h after sensitization with OVA. The mRNA expression was measured by real-time PCR. Values are presented as means ± SD (n = 6). (D) Draining LNs were collected 24 h after challenge, and total lymphocytes were stimulated with PMA/ionomycin for 6 h. IL-12 production in CD11c^+^ cells was detected by FACS after intracellular staining. Values are presented as means ± SD (n = 4). (E) WT or RAMP1-deficient BMDCs were pulsed with OVA and injected into the tail base of BALB/c mice. After 7 days, we challenged the footpad of each mouse with OVA. Values are represented as mean ± SD (n = 9). (F) The serum was collected 72 h after challenge, and the levels of OVA-specific IgG subtypes were determined by ELISA. Values are represented as mean ± SD (n = 9). *P<0.05, **P<0.01.

In order to evaluate the physiological role of CGRP in Th1 differentiation *in vivo*, RAMP1-deficient mice were immunized with OVA to induce the DTH model, which is known as a Th1 cell-mediated type IV hypersensitivity. Seven days after sensitization, mice were challenged with OVA, and then ear swelling was measured. As shown in Fig. 3A, ear swelling in OVA-induced DTH was significantly increased in RAMP1-deficient mice compared with WT mice. When total lymphocytes derived from draining lymph nodes (dLNs) of challenged mice were stimulated with anti-CD3 and anti-CD28 mAbs, increased production of IFN-γ was observed in challenged RAMP1-deficient mice (Fig. 3B). In contrast, IL-4 production was lower in RAMP1-deficient mice than in WT mice (Fig. 3B); these results indicate that Th1 responses were promoted in RAMP1-deficient mice. We next examined the contribution of DCs in the sensitization phase of DTH and Th1 differentiation. As shown in Fig. 3C and 3D, compared with those from WT mice, DCs from dLN of RAMP1-deficient mice showed a tendency to express a high level of IL-12. This observation clearly showed that CGRP physiologically regulates cytokine productions from DCs *in vivo*. To elucidate the direct effect of CGRP on DCs in the DTH model, OVA-pulsed BMDCs from WT or RAMP1-deficient mice were transferred into WT mice and mice were challenged with OVA at 7 days after transfer. As shown in Fig. 3E, footpad swelling was significantly increased in the mice who received RAMP1-deficient BMDCs compared with the mice who received WT BMDCs after being challenged with OVA. Furthermore, OVA-specific total IgG and Th1-mediated IgG2a production also increased in the mice who received RAMP1-deficient BMDCs (Fig. 3F). These results show that CGRP has the direct physiological effects in DCs both in acute innate immune responses and delayed type immune responses including Th1 cell activation and IgG production. Therefore our in vivo experiments using RAMP1-deficient mice suggest that CGRP physiologically suppresses DC function in OVA-induced DTH, and the inhibitory effect of CGRP on the inflammatory cytokine production from DCs partially explains the significant swelling of ear and footpad in RAMP1-deficient mice.

### CCR2-positive DCs Increase in DTH-induced RAMP1-deficient Mice

**Figure 4:**
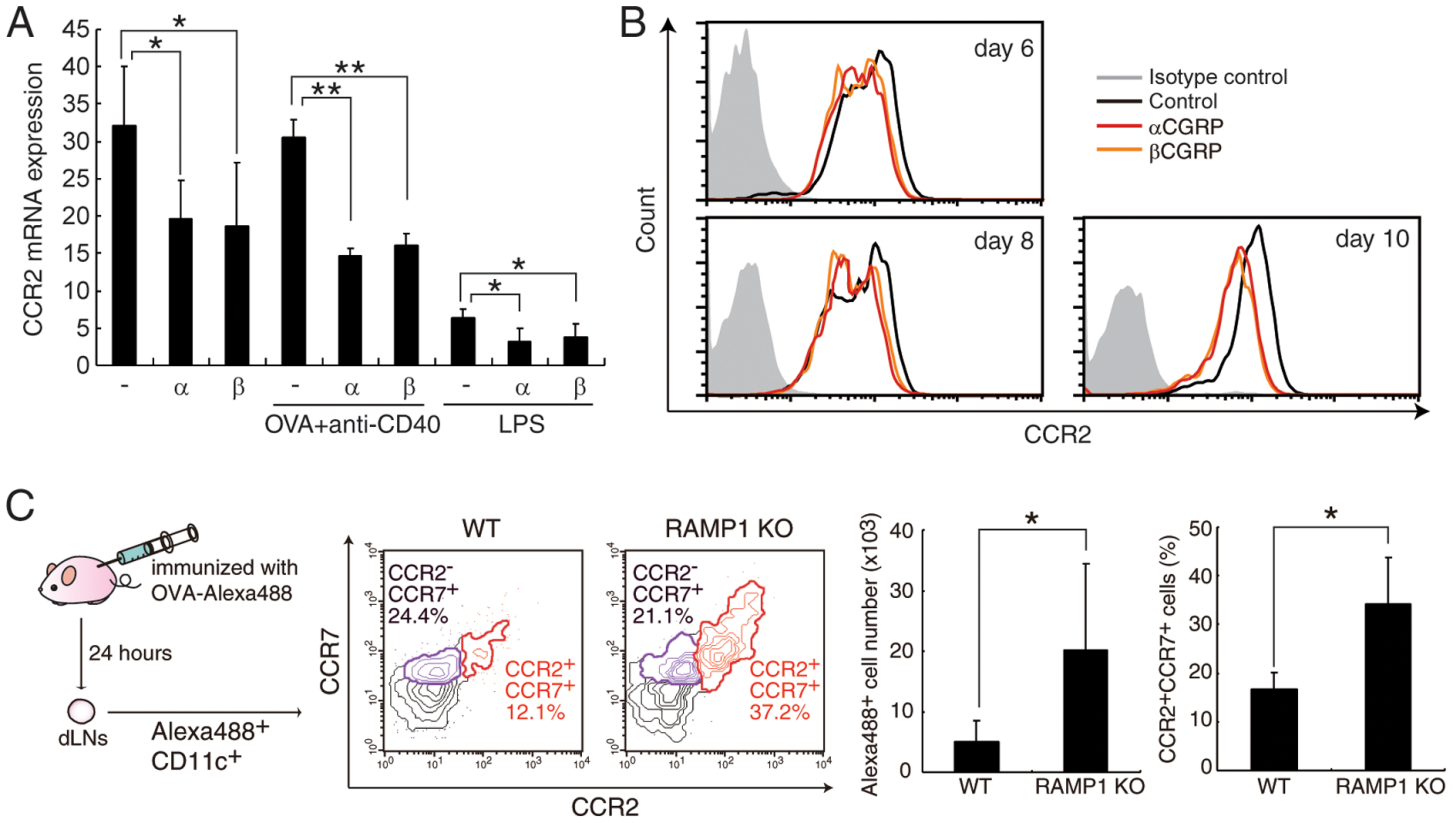
CGRP inhibits CCR2 expression *in vitro* and *in vivo*. (A) The mRNA expression of CCR2 was determined by real-time PCR using untreated-, CD40-stimulated or LPS-stimulated BMDCs. Values are presented as mean ± SD (n = 3). *P<0.05, **P<0.01. (B) BMDCs were differentiated in the presence or absence of CGRP. The expression level of CCR2 protein at each time point was measured by FACS. (C) WT and RAMP1-deficient mice were sensitized with Alexa488-labeled OVA, and migrated DCs in dLNs were analyzed by FACS. Values are presented as mean ± SD (n = 3. *P<0.05.

To further examine the effect of CGRP on DCs function, we focused on the migration of DCs in the OVA-induced DTH model because our previous study showed that CGRP inhibits migration and CCR7 expression of dermal DCs [7]. In addition, CGRP inhibited the expressions of CCR2 and its ligands (Fig. 1E and Fig. S4), and CCR2-deficient mice show reduced DC migration, Th1 reactions, and hypersensitivity [19], [20]. Therefore, the physiological effects of CGRP on the expression of CCR2 in DTH responses were examined. Consistent with a previous report [22], CCR2 expression was down-regulated in LPS-stimulated DCs on both mRNA and protein levels (Fig. 4A, and data not shown). Importantly, CGRP suppressed the CCR2 expression not only in stimulated DCs, but also in non-stimulated DCs (Fig. 4A and B). CCR2 signaling and down-regulation of CCR2 expression is considered to be one of the key events during the maturation of DCs; therefore, these results suggest that CGRP has the potential to inhibit maturation of DCs by suppressing CCR2 signaling in immature DCs.

**Figure 5:**
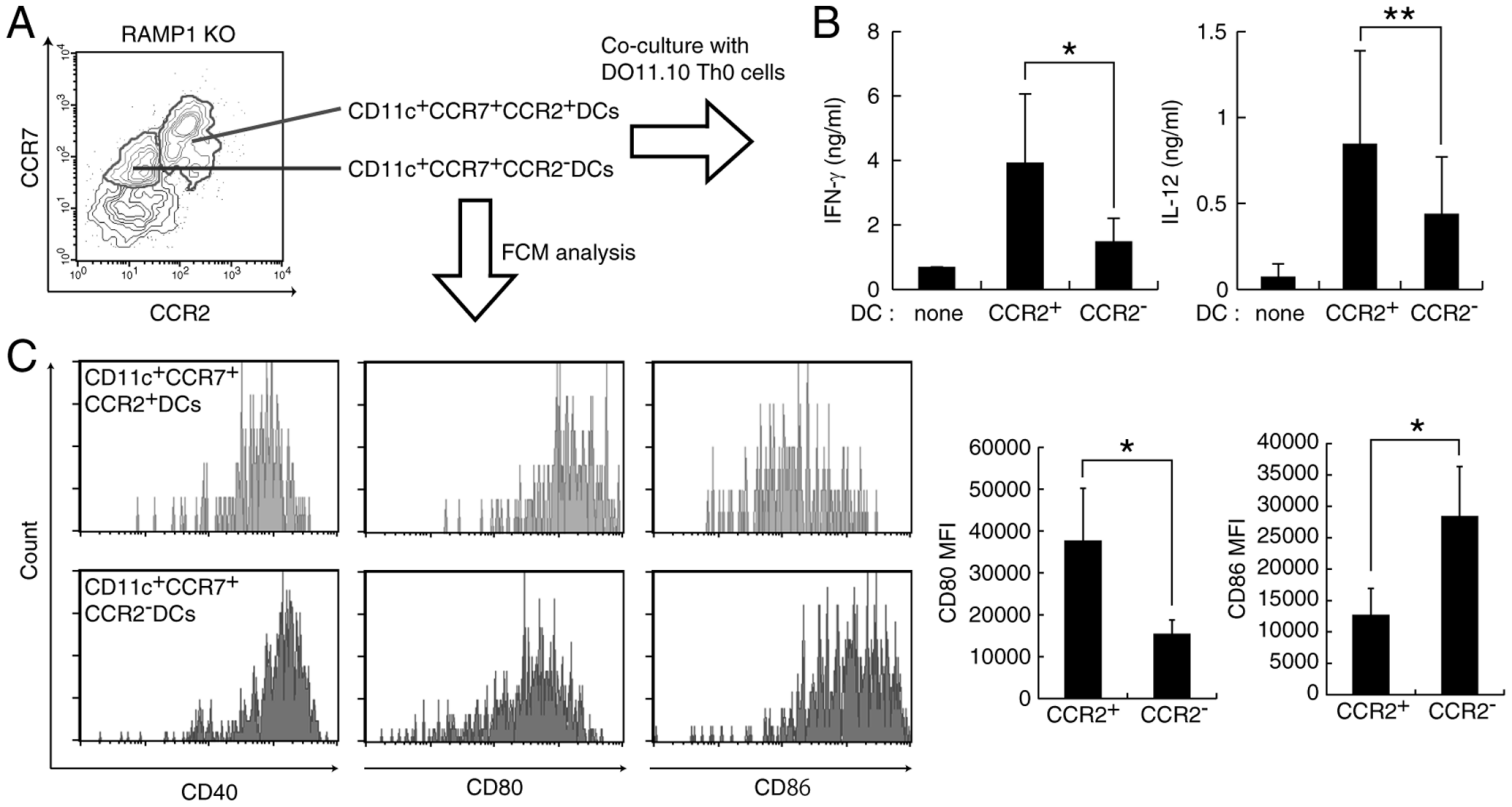
CCR2-positive DCs have a potent capacity to induce Th1 cell differentiation. (A) FACS of CCR2^+^ or CCR2^−^ DCs in OVA sensitized LNs. (B) Sorted DCs were co-cultured with DO11.10 Th0 cells for 72 h. Cytokines in the supernatant were measured by ELISA. Values are presented as mean ± SD (n = 4). *P<0.05, **P<0.01. (C) The expression of co-stimulation molecules in DCs was analyzed by FACS. Values are presented as mean ± SD (n = 3). **P<0.05.

The expression of CCR2 was further examined in migrated DCs after OVA immunization in the DTH model. As shown in Fig. 4C, migrated CCR2-expressing DCs were increased in dLN of RAMP1-deficient mice. The total number of migrated DCs was also increased in RAMP1-deficient mice, which is consistent with the decreased number of DCs migrating into dLNs in CCR2-deficient mice [23]. Next, the functions of these CCR2^+^ DCs were elucidated. CCR2+ and CCR2- DCs were separated from dLNs of OVA-immunized RAMP1-deficient mice (Fig. 5A) and co-cultured with DO11.10 Th0 cells because a large enough number of CCR2+ DCs from dLNs of WT could not be obtained. Intriguingly, the results of IFN-γ production indicated that CCR2^+^ DCs have greater ability to induce Th1 cell differentiation than CCR2^−^ DCs (Fig. 5B). The production of IL-12 was also increased in CCR2^+^ DCs (Fig. 5B), and the increased IL-12 production might explain the skewing of Th cell phenotypes toward Th1 differentiation in Th cells cocultured with CCR2^+^ DCs. Furthermore, the expression levels of costimulatory molecules also differed between CCR2^+^ and CCR2^−^ DCs. As shown in Fig. 5C, CCR2^+^ DCs showed higher expression of CD80 but lower expression of CD86 compared with CCR2^−^ DCs. Several studies indicated that CD80 costimulation induces Th1 cell differentiation but CD86 promotes Th2 cell differentiation [24]. Therefore, different expressions of costimulatory molecules might also contribute to the accelerated Th1-response in RAMP1-deficient mice.

## Discussion

The activation of DCs is mainly triggered by stimulation of pattern recognition receptors such as TLRs. Additionally, CD40 signaling cooperates with TLR stimulation to induce a synergic increase in cytokine secretion *in vivo* [25]. The intracellular signaling of these receptors has been well studied. For example, when TLR4 is stimulated, the MyD88-dependent pathway leads to activation of NF-κB in an early phase, and a later response uses the TIR-domain-containing adapter-inducing interferon-beta (TRIF)-dependent pathway, which leads to the late activation of NF-κB and IRF3 [26], [27]. On the other hand, CD40 signaling activates Akt through phosphatidylinositol 3-kinase in a ligand-dependent manner [28]. It has been shown that NF-κB is activated by Akt-dependent IκBα kinase phosphorylation [29]; therefore, CD40 also activates NF-κB in DCs [30]. These activations of NF-κB and other transcriptional factors induce cytokine production in DCs.

The activation of MAPK is another major signaling pathway in TLR-induced responses [26], [27], and it has been demonstrated that PKA-dependent p38 MAPK inhibition down-regulates IL-12 p40 transcription in macrophages and fibroblast-like cells [31], [32]. Because CGRP can also activate PKA, this pathway might control the cytokine regulation of CGRP in DCs. However, Harzenetter *et al.* reported that CGRP does not affect the TLR-triggered activation of NF-κB and MAPK, and the inhibitory effect of CGRP involves a transcriptional repressor, termed inducible cAMP early repressor (ICER) in murine BMDCs [33]. Actually, we also observed that CGRP does not affect the activation of NF-κB p65 after LPS stimulation (data not shown). Therefore, similar to the suppression of TNF-α by ICER, these results suggest that the CGRP/cAMP/PKA pathway also activates NF-κB-independent mechanisms to inhibit transcription of IL-12.

In contrast, CGRP promotes IL-10 production. In human monocytes, IL-10 production is mediated by C/EBPα binding to its promoter region, and cAMP promotes its binding [34]. Another report demonstrated that the activation of PKA induces CREB binding to the promoter region of IL-10 under TLR2 stimulation [35]. Therefore, CGRP might promote IL-10 production through cAMP/PKA-dependent C/EBPα and CREB activations. In fact, our result shows that CGRP can up-regulate IL-10 transcription without TLR stimulation, indicating that CGRP signaling induces IL-10 production independent of TLR signaling in an additive manner.

One of our new findings is that CGRP and the cAMP/PKA pathway down-regulate the expression of CCR2 and its ligand. A study of CCR2-deficient mice showed that a lack of CCR2 reduces expression of co-stimulatory molecules (CD80, CD86, and CD40) and IL-12 [18]. Therefore, these results suggest that CCR2 signaling contributes to the maturation and activation of DCs. During activation of DCs, the expression of CCR2 is down-regulated, and then CCR7 is expressed instead. Our results also indicated that LPS stimulation decreases CCR2 expression, as did previous reports [22], [36]–[39]. It is considered that the suppression of CCR2 expression allows DCs to respond to CCR7 ligands, such as CCL19 or CCL21, and perhaps down-regulation of CCR2 suppresses excess activation by CCR2 signaling in stimulated DCs. Therefore, the control of CCR2 expression seems to be one of the key mechanisms for the activation of DCs. Recent studies indicated the regulatory mechanisms of down-regulation of CCR2 in LPS stimulation in monocytes. Xu *et al.* demonstrated that LPS modulates the expression of CCR2 by deadenylation and degradation of mRNA and by internalization and degradation of CCR2 protein [38], [39]. However, it was unclear whether the regulation by CGRP overlaps with TLR-mediated regulation of CCR2 expression. Another insight into CCR2 expression showed that a transcription factor, NFAT, can induce the expression of CCR2 in DRG neurons [40]. In osteoclasts, PKA directly inhibits NFATc1 nuclear localization [41]; therefore, CCR2 down-regulation by CGRP might result from the inactivation of NFATc1 by PKA. Interestingly, CCR2^+^ migrated DCs are present in dLNs in RAMP1-deficient mice after sensitization in our study, which indicates that the absence of CGRP signaling results in the maintenance of CCR2 expression in DCs even while interacting with T cells. Because CD40 stimulation does not decrease the expression of CCR2, unlike TLR stimulation, these observations suggest the hypothesis that CCR2 signaling might contribute to the induction of Th1 cells via up-regulation of IL-12. In support of this hypothesis, our *ex vivo* coculture experiment showed that these migrated CCR2^+^ DCs produced a large amount of IL-12, highly expressed CD80, and strongly induced Th1 cells. Collectively, CGRP might control CCR2 expression in activated DCs to suppress the skew of Th1-mediated immune response.

**Figure 6:**
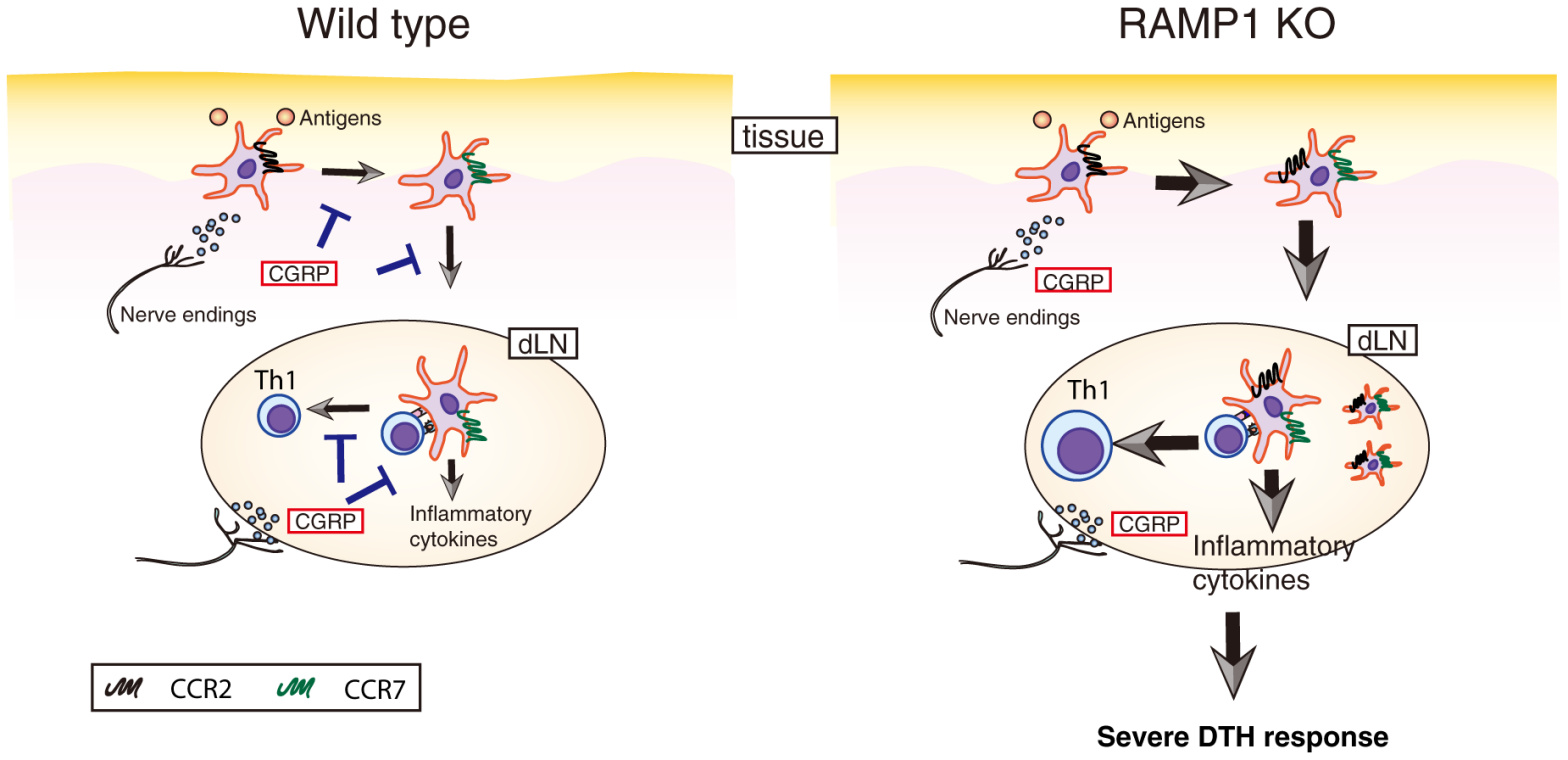
The cartoon depicts functions of CGRP in the DC-mediated immune responses. CGRP physiologically regulates DC responses as described in ‘Wild type’. In the absence of RAMP1, the increased number of migrated DCs in dLN after immunization, the unusual CCR2 expression on migrated DCs, and the higher expression of inflammatory cytokines were observed. These result in severe DTH response.

It has been reported that administration of CGRP inhibits Th1-type adaptive immunity and DTH response [42]. Our previous study demonstrated the inhibitory effects of CGRP on the DC migration and CCR7 expression using a skin inflammation model [7], but the mechanism and importance of these regulations were not defined. In the present study, the physiological role of CGRP in DTH was examined, and novel CCR2-mediated mechanisms are proposed. CCR2 signaling also contributes to the expression of CCR7 [18], and CCR2^+^ DCs showed higher expression of CCR7 than CCR2^−^ populations after OVA immunization in our experiments. Hence, it is possible that our previous observation can be explained by the effect on CCR2. Moreover, our analyses using a BMDC transfer system indicated that the direct effect of CGRP on DCs is critical in type IV hypersensitivity. Although some questions still remain, the present study indicated the physiological roles of CGRP in DC-Th cell interaction and Th1-mediated inflammation via the regulation of cytokine and chemokine signaling of DCs as summarized in Fig. 6.

## Supporting Information

Figure S1 **CGRP regulates cytokine productions by several TLR stimulations.** The concentration of IL-12 (A), TNF-α (B), IL-6 (C), and IL-10 (D) were measured by ELISA 24 hours after each TLR stimulation. All reagents were supplied at the start of culture. These data are represented as mean ± SD from three mice. **P<0.01, *P<0.05 vs. TLR stimulation only. α: 1 nM αCGRP, β: 1 nM βCGRP.
(TIF)

Figure S2 **CGRP up-regulates IL-17 production in the co-culture assay of DO11.10 Th cells and BMDCs.** DO11.10 Th0 cells and BMDCs were co-cultured in the presence of OVA and TGF-β (5 ng/ml) for 72 hours. The concentration of IL-17 was measured by ELISA. These data are represented as mean ± SD from three mice. *P<0.05 vs. control.
(TIF)

Figure S3 **CGRP regulates mRNA expression of cytokines.** BMDCs were cultured with or without LPS stimulation for 6 hours. The mRNA expressions of IL-12 p40 (A), TNF-α (B) and IL-10 (C) were measured by real-time PCR. These data are represented as mean ± SD from three mice. **P<0.01, *P<0.05 vs. control.
(TIF)

Figure S4 **The result of real-time PCR array.** The fold change of mRNA expression in the presence of CGRP was measured by real-time PCR array analysis. These data are represented as mean from three mice. ND: not detected.
(TIF)

Figure S5 **CGRP regulates cytokine and chemokine productions in DCs and macrophages.** BMDCs (differentiated by GM-CSF for 10 days) and bone marrow-derived macrophages (BMM, differentiated by M-CSF for 6 days) were stimulated with LPS. The concentration of IL-12 (A), TNF-α (B), CCL12 (C) and CCL2 (D) at 24 hours after LPS stimulation was measured by ELISA. These data are represented as mean ± SD from three mice. **P<0.01, *P<0.05 vs. LPS stimulation only. α: 1 nM αCGRP, β: 1 nM βCGRP.
(TIF)
