# Supplementary figures and images for: Calcitonin Gene-Related Peptide Regulates Type IV Hypersensitivity through Dendritic Cell Functions
Source: PLoS One. 2014 Jan 21;9(1):e86367. doi: 10.1371/journal.pone.0086367 (PMC3897726; doi:10.1371/journal.pone.0086367)

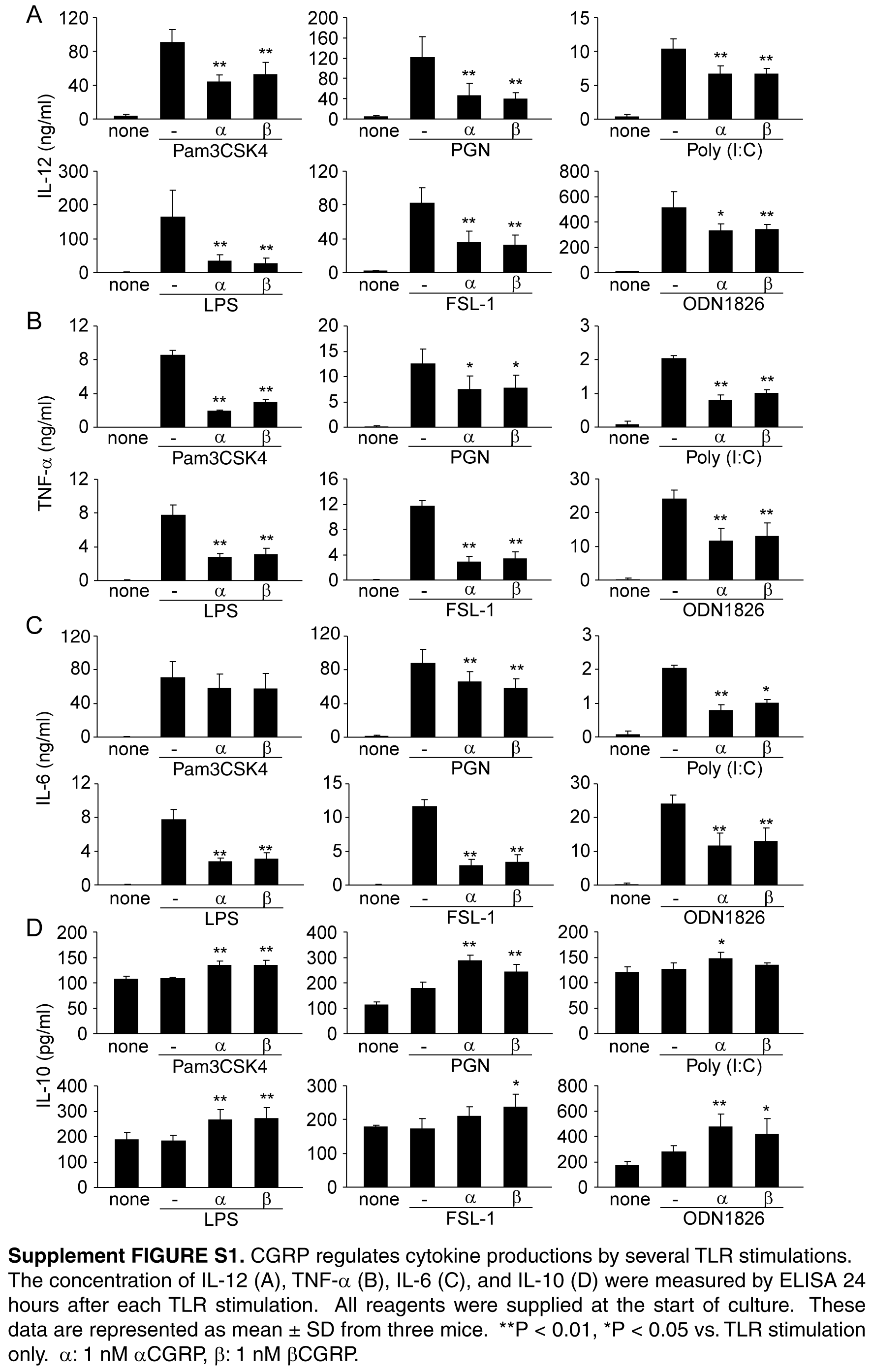

Supplement: Figure S1 — CGRP regulates cytokine productions by several TLR stimulations. The concentration of IL-12 (A), TNF-α (B), IL-6 (C), and IL-10 (D) were measured by ELISA 24 hours after each TLR stimulation. All reagents were supplied at the start of culture. These data are represented as mean ± SD from three mice. **P<0.01, *P<0.05 vs. TLR stimulation only. α: 1 nM αCGRP, β: 1 nM βCGRP. (TIF) [file pone.0086367.s001.tif]

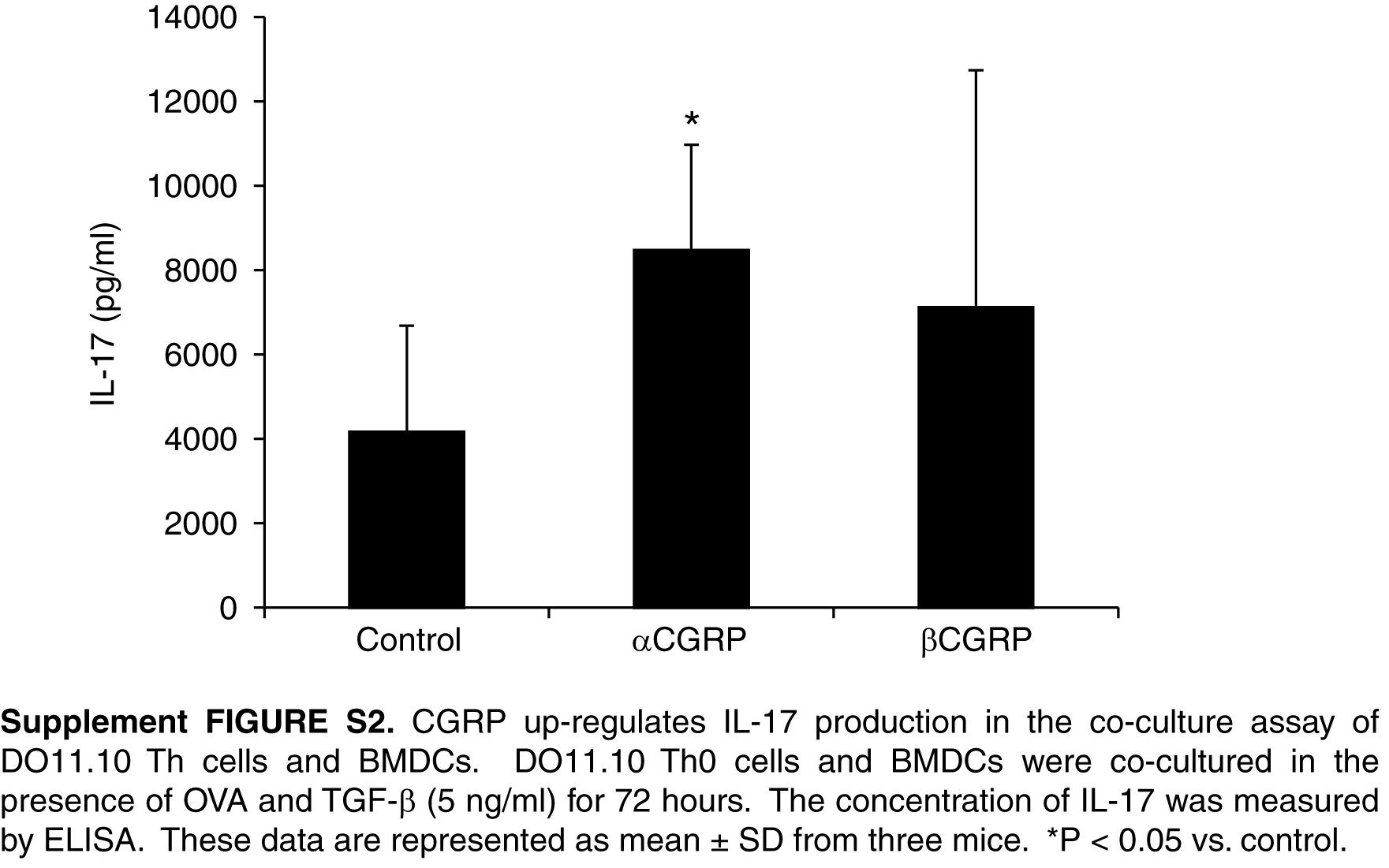

Supplement: Figure S2 — CGRP up-regulates IL-17 production in the co-culture assay of DO11.10 Th cells and BMDCs. DO11.10 Th0 cells and BMDCs were co-cultured in the presence of OVA and TGF-β (5 ng/ml) for 72 hours. The concentration of IL-17 was measured by ELISA. These data are represented as mean ± SD from three mice. *P<0.05 vs. control. (TIF) [file pone.0086367.s002.tif]

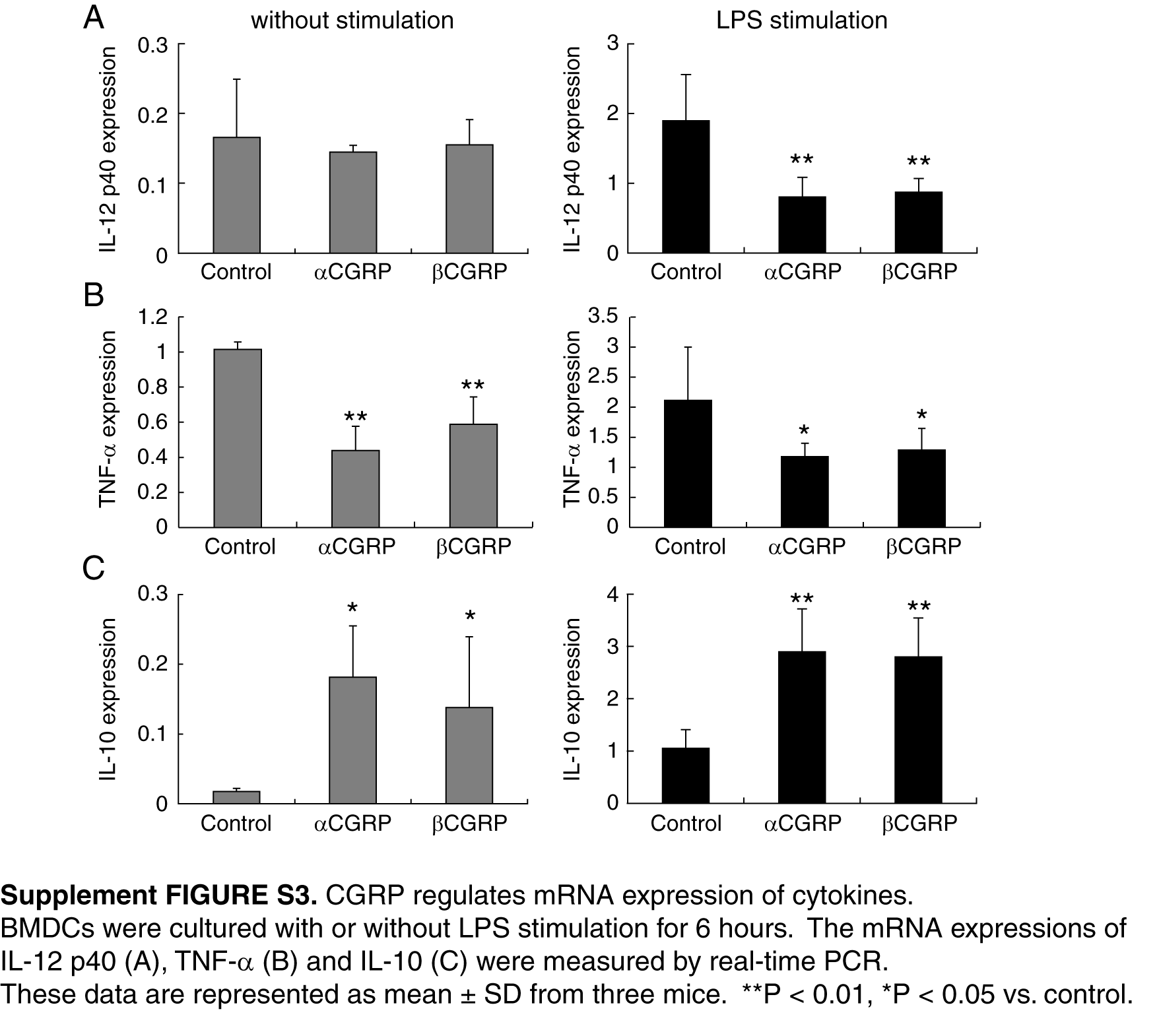

Supplement: Figure S3 — CGRP regulates mRNA expression of cytokines. BMDCs were cultured with or without LPS stimulation for 6 hours. The mRNA expressions of IL-12 p40 (A), TNF-α (B) and IL-10 (C) were measured by real-time PCR. These data are represented as mean ± SD from three mice. **P<0.01, *P<0.05 vs. control. (TIF) [file pone.0086367.s003.tif]

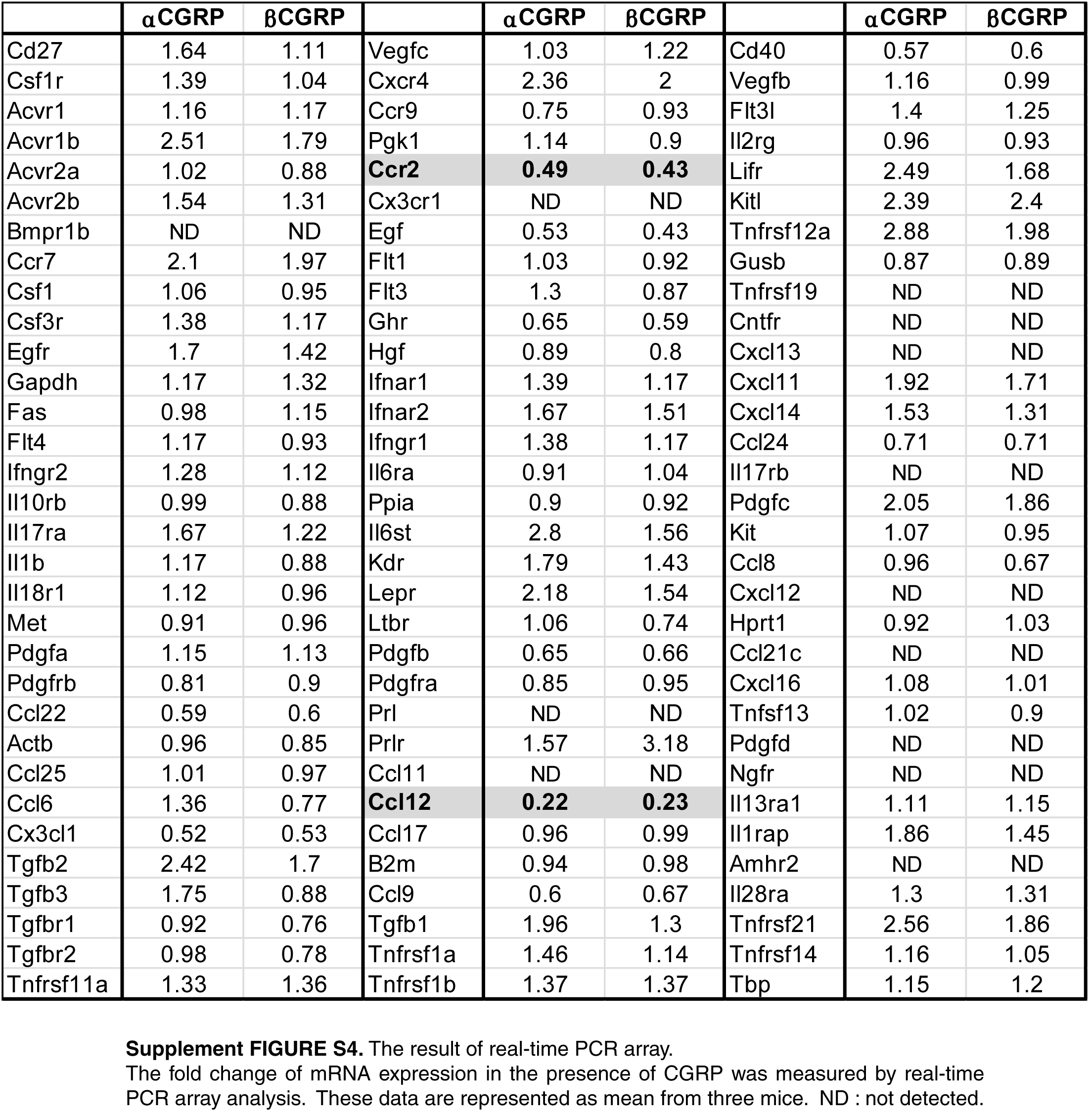

Supplement: Figure S4 — The result of real-time PCR array. The fold change of mRNA expression in the presence of CGRP was measured by real-time PCR array analysis. These data are represented as mean from three mice. ND: not detected. (TIF) [file pone.0086367.s004.tif]

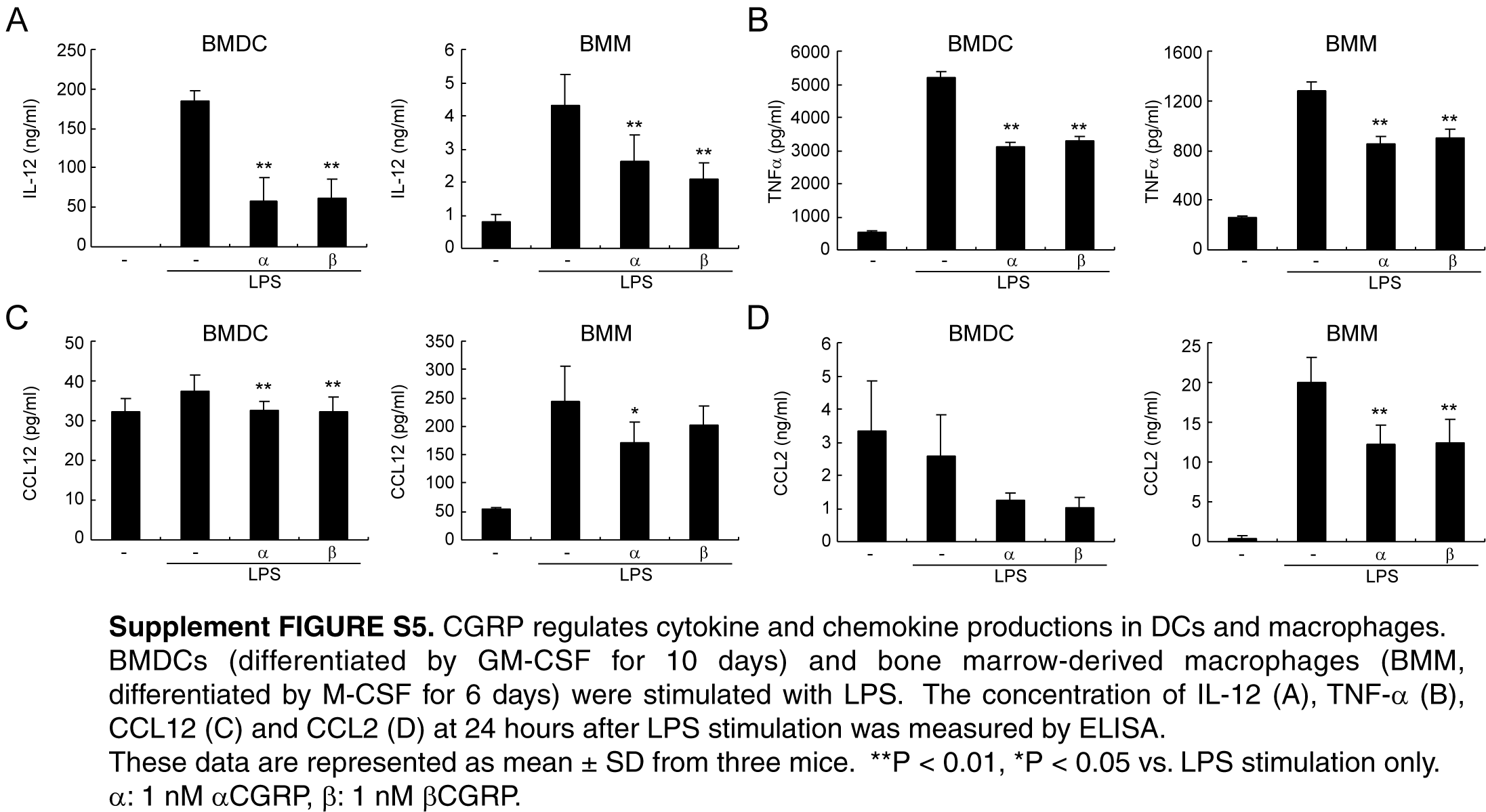

Supplement: Figure S5 — CGRP regulates cytokine and chemokine productions in DCs and macrophages. BMDCs (differentiated by GM-CSF for 10 days) and bone marrow-derived macrophages (BMM, differentiated by M-CSF for 6 days) were stimulated with LPS. The concentration of IL-12 (A), TNF-α (B), CCL12 (C) and CCL2 (D) at 24 hours after LPS stimulation was measured by ELISA. These data are represented as mean ± SD from three mice. **P<0.01, *P<0.05 vs. LPS stimulation only. α: 1 nM αCGRP, β: 1 nM βCGRP. (TIF) [file pone.0086367.s005.tif]
